# Mitochondrial Control Region Variability in *Mytilus galloprovincialis* Populations from the Central-Eastern Mediterranean Sea

**DOI:** 10.3390/ijms150711614

**Published:** 2014-06-30

**Authors:** Ioannis A. Giantsis, Theodore J. Abatzopoulos, Panagiotis Angelidis, Apostolos P. Apostolidis

**Affiliations:** 1 Laboratory of Ichthyology and Fisheries, Faculty of Agriculture, Forestry and Natural Environment, Aristotle University of Thessaloniki, Thessaloniki 54124, Greece; E-Mail: igiants@agro.auth.gr; 2 Department of Genetics, Development and Molecular Biology, School of Biology, Aristotle University of Thessaloniki, Thessaloniki 54124, Greece; E-Mail: abatzop@bio.auth.gr; 3 Laboratory of Ichthyology, Faculty of Veterinary Medicine, Aristotle University of Thessaloniki, Thessaloniki 54124, Greece; E-Mail: panangel@vet.auth.gr

**Keywords:** *Mytilus galloprovincialis*, Mediterranean, control region, translocations, sequencing, genetic diversity, Thermaikos gulf

## Abstract

The variable domain 1 (VD1) domain of the control region and a small segment of the *rrnaL* gene of the F mtDNA type were sequenced and analyzed in 174 specimens of *Mytilus galloprovincialis*. Samples were collected from eight locations in four Central-Eastern (CE) Mediterranean countries (Italy, Croatia, Greece and Turkey). A new primer, specific for the F mtDNA type, was designed for the sequencing procedure. In total 40 different haplotypes were recorded, 24 of which were unique. Aside from the two populations situated in Thermaikos gulf (Northern Aegean, Greece), relatively high levels of haplotype and nucleotide diversity were estimated for both Central and Eastern Mediterranean populations. Eight out of the 40 haplotypes were shared by at least three populations while two of them were found in all populations. Φ_ST_ and cluster analysis revealed lack of structuring among CE Mediterranean populations with the exception of those located at the Sea of Marmara and Croatian coast which were highly differentiated. Apart from the species’ inherit dispersal ability, anthropogenic activities, such as the repeated translocations of mussel spat, seem to have played an important role in shaping the current genetic population structure of CE *M. galloprovincialis* mussels.

## 1. Introduction

Marine mussels of the genus *Mytilus* constitute an economically and ecologically important group of organisms in Europe where more than 50% of their annual worldwide harvest is produced [1]. Knowledge of population genetic structure is essential for the management of all species, particularly those with cultured populations. Numerous studies have dealt with the genetic affinities of European *Mytilus* populations using a variety of molecular markers and techniques. However, the results of these studies were often controversial reflecting probably the differential resolving power of the various markers used and the individual characteristics of the examined populations. For instance, while microsatellite analyses indicated extensive gene flow across Croatian coasts [2] and potential panmixia among the mussel populations of Aegean and Ionian Seas [3], RAPD markers [4] revealed heterogeneity among Aegean populations whereas mitochondrial PCR-RFLP analyses recorded significant differentiation between Aegean and Ionian populations [5]. It is apparent that multidisciplinarity is the key-approach to reaching reliable inferences.

Mitochondrial DNA markers have generally proven to be very informative when examining genetic structure and routes of colonization in marine invertebrates [6]. However, special attention should be paid when studying population structure in species bearing the unusual system of doubly uniparental inheritance (DUI) of mtDNA [5]. The genus *Mytilus* is the taxon in which DUI has been firstly described by Zouros *et al.* [7]. According to this system, males are heteroplasmic, carrying two types of mitochondrial genomes (F and M type), inherited by both of their parents, whereas females are homoplasmic carrying only the F type inherited exclusively from their mothers. M type evolves faster and reveals higher degrees of differentiation among populations, thus theoretically it would be expected to be more efficient in population genetic analyses. However, its higher turnover rate compared to F genome, due to the tighter connectedness of the F haplotype mutational network, and its liability to invasion from the F type, a phenomenon known as “masculinization” [8], classifies the F genome as more reliable for population and phylogenetic studies [5], providing better geographical resolution [8]. Furthermore, the similar distributions of F types among males and females allow the pooling of F genomes from both genders to form one distribution of F-type haplotypes [8]. The average sequence divergence between the two genomes over the whole molecule has been reported to range from 20% [9] to 30% [10]. Nevertheless, different segments of the mtDNA molecule display different rates of divergence between the two genomes. Thereon the first part of the non-coding large unassigned region (LUR), called variable domain 1 (VD1), is among the most divergent parts [9], showing up to 80% sequence divergence and 50% difference in sequence length between the F and the M type [11].

In the present study, sequencing analysis of the VD1 domain of the F genome was applied in order to investigate the genetic relationships and population structure of *M. galloprovincialis* mussels from the Central-Eastern Mediterranean.

## 2. Results

Almost the entire VD1 region (c. 600 bp) and a small part (215 bp) of the *rrnaL* gene were aligned for the 174 specimens scored, revealing 83 variable sites that defined 40 different haplotypes, 24 of which were unique (Table 1). The length of the segments analyzed varied from 767 to 803 or to 839 bp due to a 36-bp sequence motif found in the VD1 domain that is repeated 1 or 2 times. In addition, a 6 bp deletion between the nucleotide positions 664–671 was observed in 17 individuals. The A + T nucleotide content was 55.7%, typical of a hyper variable segment as the control region [12]. The recombination analysis using the RDP3 program did not detect any evidence for recombinant variants either with or without the GenBank-obtained sequences and therefore, no haplotype was omitted from data analyses. The number of haplotypes, variable sites, haplotype and nucleotide diversities for each population is shown in Table 2, while the values of haplotype and nucleotide diversity at species level was 0.82 and 0.022, respectively.

**Table 1:** Haplotype frequencies of the populations studied. Population codes as in Table 2.

Haplotype	CHA	KAL	POR	MYT	CAN	ZAD	RAV	LIV
Mg01								0.05
Mg02	0.04							0.05
Mg03				0.04		0.09		0.05
Mg04		0.05						
Mg05				0.04		0.09		
Mg06					0.04			
Mg07					0.20			
Mg08					0.04			
Mg09			0.04		0.36		0.07	
Mg10					0.04			
Mg11					0.04			
Mg12					0.04			
Mg13					0.04			
Mg14			0.04					
Mg15			0.09					
Mg16						0.04		
Mg17				0.04		0.04		0.11
Mg18				0.04				
Mg19	0.07	0.16	0.09	0.17	0.04	0.04	0.07	0.05
Mg20	0.04							
Mg21			0.04					
Mg22						0.04		
Mg23			0.04			0.04		0.11
Mg24						0.04		
Mg25		0.05		0.09				
Mg26				0.04		0.09	0.07	0.11
Mg27				0.04				
Mg28			0.04					
Mg29						0.17		
Mg30			0.04				0.07	
Mg31							0.07	0.05
Mg32						0.04		
Mg33	0.11		0.04	0.04		0.04	0.33	0.11
Mg34						0.09		
Mg35	0.67	0.74	0.43	0.43	0.12	0.12	0.33	0.26
Mg36	0.04							
Mg37	0.04							
Mg38			0.04					
Mg39			0.04					
Mg40					0.04			

**Table 2:** A three-digit code, sample size (N), number of haplotypes (H), variable sites (Vs), haplotype diversity (Hd) and nucleotide diversity (Pi) for each population studied.

Population	Population Code	N	H	Vs	Hd	Pi
Chalastra	CHA	27	8	49	0.56	0.012
Kalohori	KAL	19	4	40	0.45	0.014
Porto Koufo	POR	23	12	62	0.81	0.023
Mytilene	MYT	23	10	51	0.79	0.023
Canakkale	CAN	25	11	47	0.85	0.017
Zadar	ZAD	23	13	54	0.93	0.024
Ravena	RAV	15	7	46	0.81	0.017
Livorno	LIV	19	11	57	0.92	0.026

**Figure 1:**
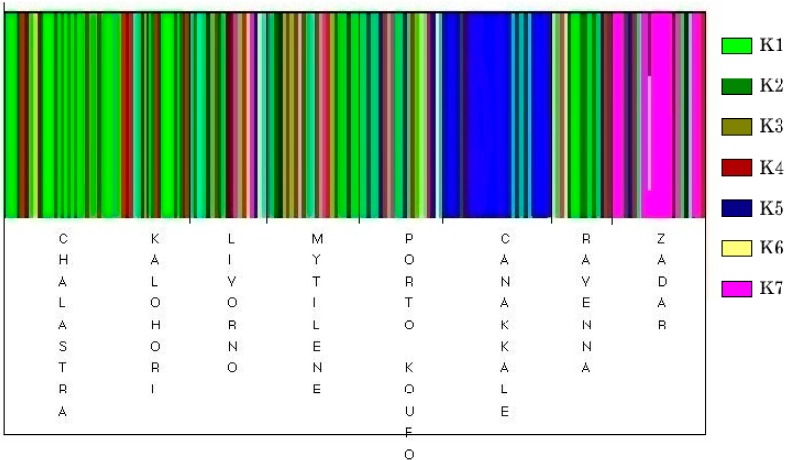
Bayesian admixture analysis of individuals. Each color represents one distinct haplogroup (cluster) while each bar represents a different individual.

Although significant genetic differentiation among all populations was depicted (Φ_ST_ = 0.12, *p* = 0.0001), this was mainly due to the samples from Canakkale and Zadar. Indeed, particularly high and significant differentiation was observed in all pairwise comparisons of Canakkale and in the most pairwise comparisons of Zadar populations (Table 3). However, omitting these populations, the overall Φ_ST_ value found was not significant (Φ_ST_ = 0.04, *p* = 0.078) suggesting homogeneity among populations and absence of geographical structure. This result was also supported by the analysis of molecular variance (AMOVA) performed on the six populations which showed that the 95.86% of the variation was attributed within populations and only 4.14% was due to variance among populations. In the cluster analysis, the highest marginal likelihood (closer to zero) was observed when the value *K* was set as 7 (*K* = 7), identifying seven distinct groups of haplotypes in optimal partition (hereafter referred as *Κ*1 to *Κ*7). With the exception of *K*5 and *K*7 groups including 21 out of the 25 specimens from Canakkale and 15 of the 23 from Zadar, respectively, no internal structuring was observed within the rest of the groups as all of them included numerous individuals from any of the remaining six populations (Figure 1).

**Table 3:** Pairwise differentiation Φ_ST_ (below diagonal) and Φ_ST_ *p* values (above diagonal). Significant *p* values after sequential Bonferroni correction for α = 0.05 are shown in bold (*p* ≤ 0.0031).

Pop.	CHA	KAL	POR	MYT	CAN	ZAD	RAV	LIV
CHA	***	0.757	0.038	0.029	**0.000**	**0.001**	0.121	0.040
KAL	0.030	***	0.072	0.160	**0.000**	**0.002**	0.123	0.121
POR	0.104	0.064	***	0.869	**0.002**	0.008	0.174	0.566
MYT	0.093	0.034	0.030	***	**0.002**	**0.002**	0.146	0.567
CAN	0.448	0.383	0.136	0.187	***	**0.003**	**0.002**	**0.001**
ZAD	0.171	0.159	0.118	0.152	0.168	***	**0.001**	**0.002**
RAV	0.047	0.046	0.031	0.031	0.334	0.199	***	0.223
LIV	0.152	0.107	0.016	0.015	0.166	0.187	0.024	***

**Figure 2:**
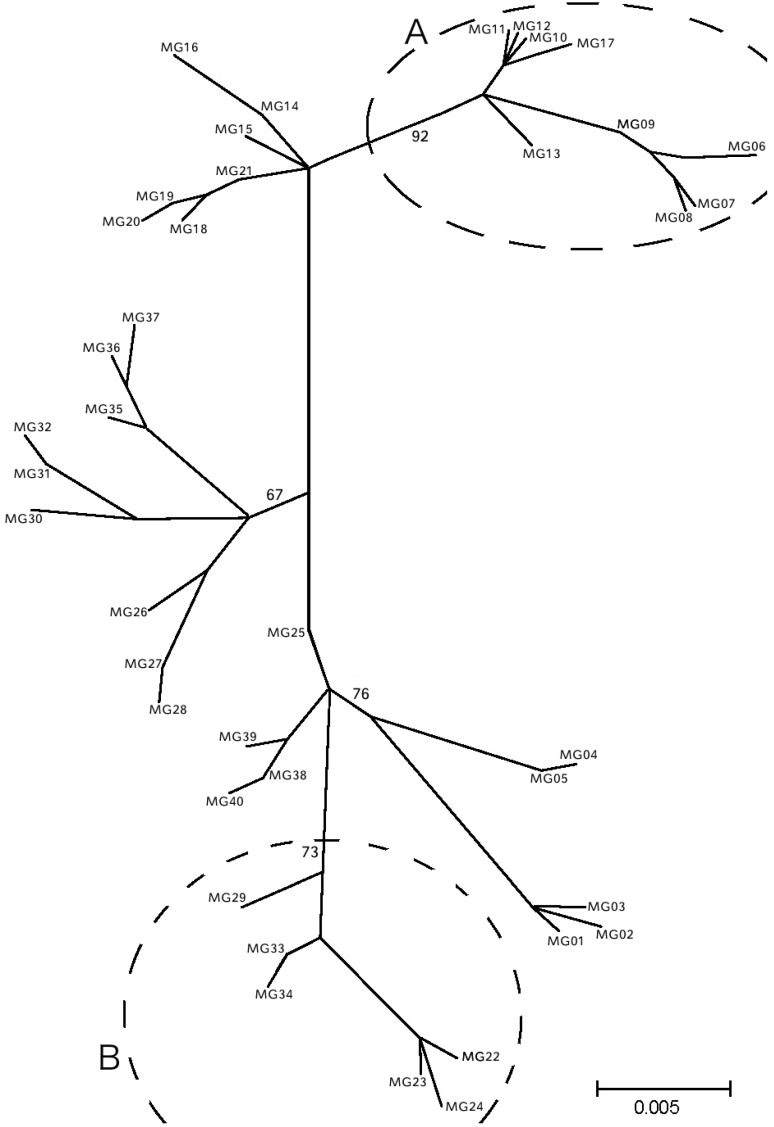
Maximum likelihood dendrogram of the haplotypes found. Group A and B consist mainly from Turkish and Croatian populations, respectively. Population codes as listed in Table 2. Bootstrap values greater than 60% are demonstrated on the branches.

Evolutionary relationships among haplotypes are shown in the ML dendrogram (Figure 2). Eight out of the 16 non-unique haplotypes were shared by at least three populations while two of them were present in all populations (Table 2 and Figure 1). Consistent with Φ_ST_ and BAPS results, the majority of the Turkish and the Croatian haplotypes were clustered in separate branches supported by high bootstrap values (group A and group B, respectively). The remaining central branches were composed by haplotypes from all the other populations (Figure 2).

## 3. Discussion

The 36-bp motif found within the VD1 region has probably resulted from replication slippage [9] and had already been described in previous studies [13,14]. However, this is the first time that it has been found two times in the same individual, *i.e.*, in one specimen from Porto Koufo, whereas it was found in at least one individual of each population examined. Worth noting that Smietanka *et al.* [13] found it only in Mediterranean and Black Sea samples, while Luis *et al.* [14] reported its presence in Atlantic mussels as well.

**Figure 3:**
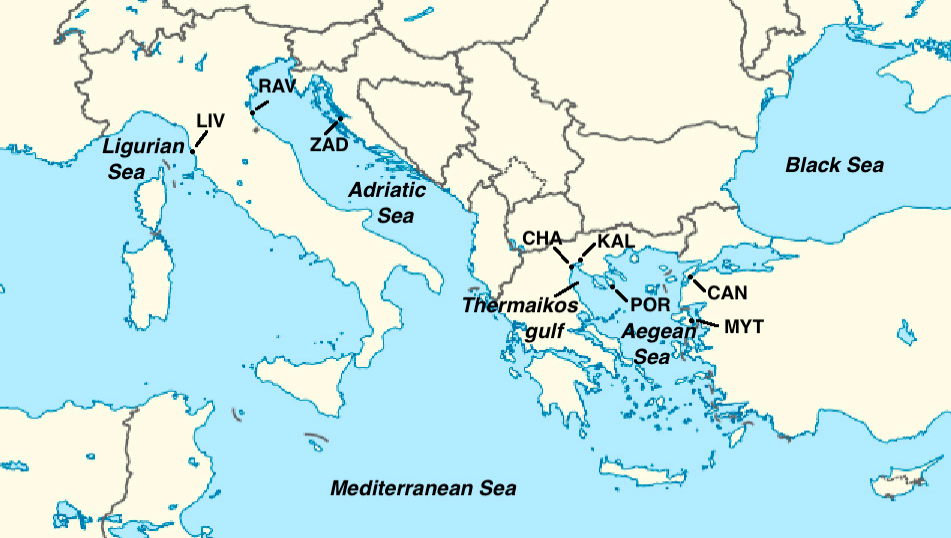
Geographical locations of the analyzed populations. Population codes as listed in Table 2.

The mean values of haplotype and nucleotide diversity recorded in this study were rather lower than those reported in previous mtDNA studies of European *M.* *galloprovincialis* populations [8,14,15]. However, this discrepancy has principally arisen from the decreased values observed in the populations of Chalastra (cultured) and Kalohori (wild), located in the north Thermaikos gulf (Table 2 and Figure 3), which exhibited far lower genetic diversity than the other populations examined. Notably, no such reduction had been previously observed in microsatellite analyses [3]. A possible explanation for these low values relies on the oceanographic conditions prevailing in the gulf [16], which might hamper the mussels from this area to renew their gene pool with spat from Aegean Sea, together with the genetic markers used. Since the effective population size of the F-mtDNA is smaller that of nuclear DNA, F-mtDNA diversity will be more sensitive to population reductions and potential bottlenecks compared with nuclear diversity. Such reductions resulting in gene pool impoverishment, may have been caused by intense harvesting and repeated translocations of spat from Chalastra to other mussel farms throughout Mediterranean coasts (see below), as well as by the commercial harvest of mussels from the area of Kalohori.

Although none of the haplogroups observed was region-specific, *K*5 and *K*7 (Figure 1) showed a clear differentiation of the Turkish and Croatian samples. This was also supported by all pairwise comparisons of these populations (Table 3) as well as by the ML dendrogram (Figure 2). The genetic distinctiveness of mussels from these regions could be attributed to oceanographic conditions and topographic peculiarities prevailing at these areas [3]. Indeed, the narrowness of Dardanelles whereby low salinity sea currents flow towards the Aegean Sea [17] and the numerous islands that surround the coasts of Croatia in combination with the cyclonic circulation of the Eastern Adriatic [18] probably constitute physical barriers to gene flow in Canakkale and Zadar, respectively. The cluster analysis classified the remaining populations into five groups that lacked internal structuring; each group contained individuals from all six populations. In addition AMOVA and Φ_ST_ analyses suggested homogenization among these populations.

The high dispersal potential of marine mussels of the genus *Mytilus* is expected to result in high gene flow among populations which may lead to genetic homogenization on a wide scale. However, in some cases, gene flow can be constrained by biological, physical and ecological factors. The topography and oceanographic conditions of Aegean Sea (East Mediterranean) and the Siculo-Tunisian strait (Central Mediterranean) have been described as transition areas that can cause population genetic differentiation between Aegean—Adriatic and Adriatic—Ligurian Seas, respectively, in numerous marine organisms [5,19,20,21]. Therefore, the observed homogeneity among mussel populations originating from the aforementioned basins is rather surprising as these would be expected to exhibit a degree of differentiation rather than homogenization. Apart from the species high dispersal ability, human mediated dispersal, *i.e.*, transplantations of spat for aquaculture purposes and transport of ships ballast water, may have also played an important role in overcoming the barriers that prevent mass gene flow between these basins and consequently may have contributed to the genetic homogeneity among CE Mediterranean populations. Transplantations have influenced the genetic composition of native mussel populations in several areas *i.e.*, along Chilean [22] and northwestern European coasts [23]. In Greece, spat from Chalastra, which serves as the main culture area in Aegean Sea, has been repeatedly translocated to other mussel farms throughout Greek, Italian and French coasts for ongrowing purposes [24]. Moreover, aged mussels are often translocated alive for sale far away from their farming units, and immerged in sea water before their consumption. During this short immersion life stage, some mussels can release genetic products and promote the dispersion of the genetic materials in the new marine environment. However, since most spat translocations are unrecorded and no official information about them exists, it is very difficult to estimate with confidence their contribution to the observed genetic homogenization.

## 4. Experimental Section

In total, 174 individuals of *M. galloprovincialis* were analyzed. These were collected in the period 2009–2011 from eight sampling sites, located in four different countries from CE Mediterranean (Figure 3 and Table 2). In the area of Chalastra mussels sampled in a long line farm, while all the rest derived from wild stocks. Total DNA was isolated from mantle tissue of each individual, applying the standard CTAB protocol of Hillis *et al.* [25].

The noncoding large unassigned region (LUR) of the mitochondrial genome was amplified in each tissue sample using the primers UNFOR1 and UNREV1 [9], located in the *rrnaL* gene (as described by Mizi *et al.* [26]) and in the tRNA-Tyr, respectively. The applied amplification conditions and technical procedures were according to Cao *et al.* [9]. PCR products were checked in 1.5% agarose gel electrophoresis stained with Midori Green DNA stain (NIPPON Genetics Europe GmbH, Düren, Germany). Given that the set of primers UNFOR1—UNREV1 amplifies the LUR of both F and M mitochondrial DNA genomes, two electrophoretic bands were produced in males whereas one in females (for details see Cao *et al.* [9]). In addition to these two types of mtDNA, *M. galloprovincialis* populations may occasionally host a third mitochondrial genome (C genome, Venetis *et al.* [27]), that is paternally transmitted. The LUR of C genome is more than threefold larger compared with F or M and it is located between the *rrnaL* gene and the tRNA-Tyr; it could be, therefore, amplified by the primers UNFOR1—UNREV1. However, in our case, no specimen with a third band was observed among the analyzed samples.

The successfully amplified products (from both males and females) were purified with the NucleoSpin PCR Clean-up Kit (Macherey-Nagel, Düren, Germany) to remove excess nucleotides, primers, enzymes and other impurities, following the manufacturer’s protocol. For the sequencing reactions, a new primer, MGF (5'-GCA AAC ACT ATT TAA GTG TA-3'), specific for the female mtDNA molecule of the VD1 domain in *M. galloprovincialis* mitochondrial F genome (GENBANK accession number: AY497292.2) was designed. Purified samples were sent to VBC-Biotech (Vienna, Austria) for sequencing. All sequences were aligned using the software BioEdit version 7.1.9 [28] and were deposited in the GENBANK database under the following accession numbers: KJ802790-KJ802829.

Since recombination between paternal and maternal lineages, as well as among female mtDNA haplotypes is possible in *Mytilus* [11,15], the Recombination Detection Program (RDP) version 3 [29] was used to detect potential recombination signals that could affect the genetic analyses. RDP is also capable to identify a “masculinized” F type (M^f^) segment that has been paternally inherited. Hence, two additional haplotypes, obtained from complete M and F mitochondrial *M. galloprovincialis* genomes in GenBank (AY363687 and AY497292, respectively) were included in the data set and the step-down correction method for significance was applied using the default settings.

Haplotype and nucleotide diversities were estimated for each population using the software DnaSP version 5.0 [30]. Genetic differentiation between pairs of populations and overall data was investigated through Φ_ST_ analyses using Arlequin version 3.5 [31]. Throughout the analyses, corrections for simultaneous multiple comparisons were applied using sequential Bonferroni correction [32]. Furthermore, possible genetic structure of populations was investigated by the software BAPS 5.4 [33] which groups genetically similar individuals into homogenous clusters applying a Bayesian approach. Cluster analysis was carried out several times performing 10 replicate runs in order to find the posterior optimal clustering. Phylogenetic relationships among all haplotypes were depicted by a maximum-likelihood (ML) diagram constructed by the program MEGA version 5.05 [34]. Sequence repeats and duplications were considered as one mutational step (one point mutation) in all structure analyses conducted.

## 5. Conclusions

Mitochondrial DNA variation revealed genetic homogenization of mussel populations in the biggest part of central-eastern Mediterranean. Apart from the species’ high dispersal ability, anthropogenic activities may have played an important role in shaping the current structuring of Mediterranean mussels. On the other hand, there are still genetically differentiated populations such as those of the Croatian coasts and the Sea of Marmara. Therefore, future management actions should be carefully designed, especially in localities harboring distinct native populations.
